# Effects of statins on mitochondrial respiration and outcome during experimental sepsis

**DOI:** 10.1186/cc11711

**Published:** 2012-11-14

**Authors:** J Morel, I Hargreaves, B Bollen-Pinto, D Brealey, J Backman, A Dyson, M Singer

**Affiliations:** 1 CHU Saint Etienne, Saint Etienne, France; 2 Neurometabolic Unit, National Hospital for Neurology and Neurosurgery Queen Square, London, UK; 3 Bloomsbury Institute of Intensive Care Medicine, University College of London, UK; 4 University of Helsinki and HUSLAB Helsinki University Central Hospital, Helsinki, Finland

## Background

Statins target several mechanisms involved in the pathophysiology of sepsis, leading to their consideration as an adjuvant therapy. Ubiquinone is an important mitochondrial antioxidant and constituent of the electron transport chain. Ubiquinone production is inhibited by statins, whereas sepsis itself also affects mitochondrial activity. The impact of statins on mitochondrial function in sepsis has not been previously explored.

## Methods

Sepsis was induced in instrumented, awake, male Wistar rats by i.p. injection of faecal slurry. Fluid resuscitation was provided by continuous intravenous infusion. Simvastatin 20 mg/kg bd was administrated by gavage commencing either 3 days pre-sepsis (pre-treatment) or from 6 hours post-sepsis (post-treatment). A control group received only vehicle but no active drug (vehicle). Survival was assessed at 72 hours (16 per group). In a second set of experiments, rats were sacrificed at 24 hours post-sepsis (seven per group) for measurement of: plasma biochemistry and simvastatin acid; heart and muscle ubiquinone levels by HPLC; and oxygen consumption on permeabilized soleus muscle fibers in a Clark electrode chamber. A fourth group of naive animals was also used as healthy controls. Statistics were performed using the Wilcoxon test and repeated-measures ANOVA and *post hoc *Bonferroni test.

## Results

**Figure 1:**
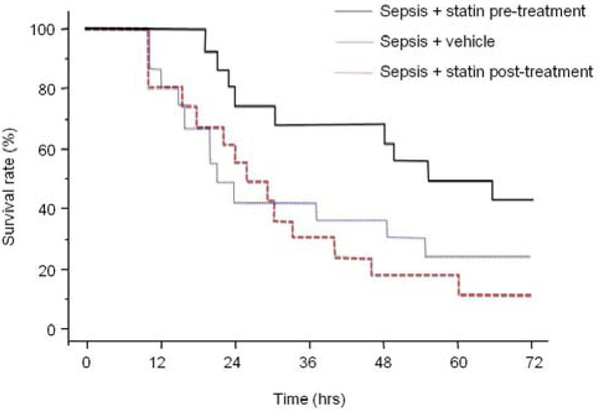
**Seventy-two-hour survival curves**.

Survival at 72 hours (Figure 1) was 43.7%, 25% and 12.5% for pre-treatment, vehicle, and post-treatment groups, respectively (*P *< 0.05). At 24 hours post sepsis, ubiquinone was significantly decreased only in hearts taken from statin pre-treated animals (706 ± 222 vs. 1,217 ± 269 pmol/mg protein for vehicle hearts). Plasma simvastatin acid levels were significantly increased in statin pre-treated animals (41 ± 3 ng/ml) compared with naïve animals receiving the same dose (1 ± 0.4 ng/ml). Compared with vehicle-treated animals, statin pre-treatment resulted in significant decreases in urea and creatinine, and a trend towards improvement for liver enzymes and creatine kinase. Plasma lipid levels were not significantly affected by statins given either before or after the onset of sepsis. Mean values of muscle oxygen consumption were 11.9 ± 5.3, 14.4 ± 2.9, 12.1 ± 1.5 and 5.9 ± 2.4 pmol/ml/second/mg for naïve, sepsis + post-treatment, sepsis + pre-treatment and sepsis + vehicle groups, respectively. The significant reduction in muscle oxygen consumption seen in the sepsis + vehicle group was prevented in both groups receiving simvastatin.

## Conclusion

This study confirms the beneficial effect of statins when given before the onset of sepsis, and this appear to be independent of its lipid-lowering property. This beneficial effect is likely to be multifactorial but could be attributed in part to a protective effect on mitochondrial respiration.

